# Understanding the U.S. Economy for Racial Healing

**DOI:** 10.1089/heq.2022.29020.aus

**Published:** 2023-01-20

**Authors:** Algernon Austin

**Affiliations:** Center for Economic and Policy Research, Washington, District of Columbia, USA.

This article will present a very short discussion of the importance of economics to understanding and eliminating racial hierarchy. Its examples are primarily based on the African American experience. Because of its short length, many issues, groups, histories, and experiences will not be discussed. Readers should take the principles presented in this study and apply them to their specific concerns.

## Racist Ideology Is Shaped by the Economy

Many people think of racism as an irrational hatred of other racial groups. Although this may accurately describe the views and actions of particular *individuals*, it is not helpful for understanding how racial beliefs and practices help to build and maintain the structure of racial inequality in a society.

Consider the fact that white racists today would not be in favor of bringing hundreds of thousands of Africans to the United States, nor smuggling them into the country if it is not legal to do so. But in antebellum America, this is exactly what white racists did. Slaveholders imported hundreds of thousands of Africans at considerable expense, and when the slave trade was made illegal, occasionally, they would smuggle Africans into the country.^1^ Racism as hatred would lead us to expect that white racists would bar Africans from the United States, but this is the opposite of what occurred.

Racist ideas and practices help to structure American society by being in dialogue with the economy of the society. The dominant racist ideologies of a society economically reliant on racial slavery will justify and support racial slavery. The economy of American society no longer rests on racial slavery, so there are no longer ideologies justifying the importation of Africans.

A key part of racism is the creation of myths to justify economic hierarchies. Racial slavery was a profitable institution for slaveholders. When slavery was challenged, the historian Peter Kolchin reports, “Southern spokesmen responded with elaborate arguments in defense of slavery, including pseudoscientific demonstrations that blacks were unfit for freedom, reminders that the nation's economic well-being depended on slave labor, assertions that the Bible itself sanctioned the enslavement of the ‘sons of Ham,’ and claims that slavery produced a more humane and harmonious social order than the exploitative free-labor system.”^2^ The desire to profit from racial slavery encouraged the development and dispersal of ideas to justify it. Looking at the broad history of racist ideologies in America, the historian Mia Bay notes that one sees “ever-changing intellectual rationalizations” to justify racial economic hierarchy.^3^

Today, we are seeing a growing concentration of wealth among the rich in the American economy. In 1982, *Forbes* magazine first published its list of the richest Americans. The man who topped the list was worth $5.8 billion adjusted for inflation.^4^ Today, that amount of money is not enough to put someone in the top 100 richest Americans. Elon Musk was recently listed as the richest man with a net worth of $219 billion.^4^ The rich have gotten a lot richer.

The rich have gotten richer due to a host of labor, tax, trade, and finance policies that have facilitated the upward distribution of income.^5^ Consequently, it is harder for average Americans to maintain their standard of living and even harder for them to be upwardly mobile. The economist Raj Chetty and his colleagues find that “more than 90% of children born in the 1940s grew up to earn more than their parents,” but “[t]oday, only half of children grow up to earn more than their parents.”^6^

For the economy to continue benefitting the rich, it is important that this growing class inequality does not receive too much public attention and analysis. As the rich have gotten richer, average white Americans have been encouraged to blame their economic stress and problems on people of color as opposed to the economic elites who are actually profiting. The scholars Michael I. Norton and Samuel Sommers find that white people believe that American society has become more biased against white people over time and less biased against black people over the same period.

In their research, white people now say that there is more anti-white bias in society than anti-black bias.^7^ Other research has found that about 40% of supposedly non-racially-biased white people believe that white people experience as much or more racial discrimination than black people.^8^ A recent survey found that a majority of whites believe that there is discrimination against white people in American society.^9^ White people believe that they are one of the primary victims of racism.

The white man, Tim Hershman of Akron, Ohio, captures some of these views well. He states, “If you apply for a job, they seem to give the blacks the first crack at it, and, basically, you know, if you want any help from the government, if you're white, you don't get it. If you're black, you get it.”^9^ Hershman is correct to feel that there is something wrong with the American economy. For example, from 1973 to 2021, the real average wage for white men with a high school diploma *declined*.^10^ In earlier periods of American history, the wages of white male high school graduates would have *increased* significantly over a similar length of time. What no one is telling people like Hershman is that the average wage for black male high school graduates is declining also.^10^ In addition, the average wage for white male high school graduates is still higher than the wage for black male graduates.^10^

Hershman's comments also seem to be referencing affirmative action in employment, which is often mischaracterized as “racial preferences” or “reverse discrimination” by opponents to the program. Affirmative action in employment is needed because *there is a strong white preference in the American labor market*. When researchers present black and white job candidates with equivalent qualifications to employers, they consistently find that employers prefer the white candidates.^11^ In one shocking study, white men *with* a criminal record had similar odds of receiving a positive response from employers as black men *without* a criminal record.^12^ Affirmative action is needed to counteract the white preference in the labor market and to help to move us to equal opportunity.

Unfortunately, affirmative action in employment has been designed to be a weak program and it is weakly enforced. It is extremely rare for firms to be subject to strong sanctions for violating affirmative action guidelines.^13^ A recent study of 108 firms found that some firms that were supposed to be following affirmative action guidelines were among the most discriminatory firms against black people.^14^ Apparently, because the program and its enforcement are so weak, firms that discriminate against black job applicants can still be deemed to be in compliance with affirmative action.

It is a myth that black people have any economic advantages over white people. The black unemployment rate has been roughly twice the white unemployment rate for the entirety of Hershman's working life.^15^ The black poverty rate has been more than twice the white rate for the entirety of his working life also.^15^ On every economic measure, black people are worse off than white people, yet more and more white people are convinced that black people have tremendous economic advantages that they do not. As long as white people are convinced that people of color are the source of their economic problems, they will never address the real issues, and they will never be able to stop the upward flow of income to the rich. The false racial ideas spread to people like Hershman serve to enable increasing class inequality.

## Much of Racial Stratification Is Class Stratification

A significant part of what we understand as racial differences are or stem from class differences. If one looks at specific economic measures, it is often the case that racial hierarchies are clearly visible. For example, the Census Bureau's Supplemental Poverty Measure for 2020 (which many analysts consider to be superior to the Official Poverty Measure) shows the non-Hispanic white population with the lowest poverty rate and the black population with the highest. The Latino population has the second highest rate, and the Asian American population has the third highest.^16,17^ Other economic measures are stratified similarly.

When people refer to black neighborhoods as the “ghetto” or the “streets” or the “hood,” they are referring to the class makeup of the neighborhood as much as the racial makeup. A black middle- or upper-class neighborhood far removed from the problems associated with poverty would not be considered the “ghetto,” the “streets,” or the “hood.” However, because black people are so strongly and consistently disadvantaged economically, there are very few black neighborhoods that are removed from the problems associated with poverty.^18,19^ The black middle class is much more exposed to the black poor than the white middle class to the white poor.^20^

In a capitalist economy, one's ability to obtain economic resources is extremely important to one's quality of life and life opportunities. For example, one's life expectancy is positively correlated with one's socioeconomic status.^21,22^ American society limits black economic opportunities, and this limitation profoundly shapes black life in a wide variety of ways.

## Asian Americans and American Racial Economic Hierarchies

Asian Americans are often misused to argue that America is post-racial. For this reason, it is useful to touch on a couple of issues regarding this population. The situation of Asian Americans is too complex for an adequate treatment in this brief document. Readers are strongly encouraged to go beyond this document and do some study of Asian American history and the sociology of Asian Americans and racial stratification if they are not already familiar with these issues. The book *The Asian American Achievement Paradox* by the sociologists Jennifer Lee and Min Zhou would be a good place for readers to start.

As mentioned earlier, Asian Americans have a higher supplemental poverty rate than non-Hispanic whites, but they also have a higher median household income.^23^ The Asian American population is diverse in many respects, but they tend to be of a higher socioeconomic status. More than half of the Asian American labor force has a bachelor's or advanced degree compared with about a third for the rest of the U.S. labor force.^24^ Much of the apparent economic success of Asian Americans stems from this high educational attainment.

The root of Asian American high educational attainment is the post-1965 bias in the U.S. immigration process in favor of Asian immigrants with college degrees.^25^ For example, in their analysis, Lee and Zhou found that half of Chinese immigrants had a bachelor's or higher degree, but only 4% of adults in China were similarly educated.^26^ The immigration process does not lead to a random sample of the Chinese population coming to the United States, it disproportionately selects Chinese immigrants with college educations. Parent's educational attainment is a powerful predictor of a child's educational attainment.^26^ Thus, the children of highly educated Asian immigrants are more likely to be also highly educated and, therefore, relatively economically successful.

## Economic Inequality Contributes to Racial Inequality

Economic inequality has increased tremendously over recent decades. In 1967, the bottom 60% of households ranked by income earned only about 30% of all the income—half their population share. The richest 5% of households earned more than three times their share of the population—17.2%. There was significant income inequality in 1967, but it has gotten considerably worse today. In 2020, the bottom 60% only earned 25% of all income—less than half their population share. The richest 5% earned nearly as much as the bottom 60–23%—nearly five times their population share.^27^ Because the black population is disproportionately lower income, they are over-represented on the losing side of this widening income inequality.

The economist Valerie Wilson conducted an analysis of black–white wage inequality that compares both the racial component and the class component. Looking at wage inequality from 1979 to 2015, she finds that eliminating the racial inequality component would increase black wages by about $5 per hour. Eliminating the class component would increase black wages by over $12 per hour.^28^ In other words, class inequality cost black workers more than twice as much as the racial wage inequality. Of course, the ideal situation is to address *both* racial and class inequality. But it is important to remember that if we ignore class inequality while pursuing racial equality, we will still leave black people significantly economically disadvantaged.

## Americans of All Races Prosper from Racial Equity

Racial discrimination and economic inequality ultimately hurt the majority of people of all races. It only enriches economic elites. The economic policy expert Heather McGhee documents this in her book *The Sum of Us: What Racism Costs Everyone and How We Can Prosper Together*. For example, she reports that slavery underdeveloped the South. “[C]ounties that relied more on slave labor in 1860 had lower per capita incomes in 2000,” she states, and “[w]hen slavery was abolished, Confederate states found themselves far behind northern states in the creation of the public infrastructure that supports economic mobility, and they continue to lag behind today.”^29^

America today is not being held back by slavery anymore, but it is being held back by a host of labor, tax, trade, and finance policies that have accelerated economic inequality.^5^ Income given to the rich is often less economically productive than income given to the poor.^30^ Imagine that a black family living in poverty receives an extra $1000. This family will likely spend all of it, and that $1000 will circulate and generate additional economic activity. For example, the convenience store near that black family's home might receive a portion of that $1000 in exchange for goods, and, in turn, the store owner would use that income to buy more goods and to help pay a staff person's wages.

What happens in the American economy if Elon Musk receives an additional $1000? Absolutely nothing. There is nothing that he wants for which an additional $1000 would make a difference. Musk's $1000 is much less economically productive than the $1000 going to the poor black family. As income inequality has increased in the United States and shifted more income to the rich, it has slowed U.S. economic growth and widened racial economic inequality.^30^

Racial segregation has also been shown to have a negative effect on economic growth, and racial integration a positive one. After analyzing metropolitan area segregation and economic growth, the economist Huiping Li et al. conclude, “Residential segregation is thus detrimental to the welfare of all the people, both the poor and non-poor, in central cities and suburbs.”^31^ Mark Zandi, the chief economist at Moody's Analytics, and his colleagues concur. Analyzing segregation at the county level, they find a positive relationship between racial integration and economic growth. They conclude that “the more racially integrated our society, the stronger our economy.” They add, “Indeed, if communities across the country were to more fully integrate racially so that they were comparable with the nation's most integrated communities,” it “would be an economic game changer.”^32^ The racial discrimination and political divisiveness that underlies racial segregation are the likely causes behind the weaker growth from racial segregation.^33,34^

Racism also prevents the country from protecting itself from economic threats and crises. McGhee gives the example of the predatory subprime mortgage crisis that caused the Great Recession. She states, “Bank regulators and federal policymakers were well aware of what was happening in communities of color, but despite pleas from local officials and community groups, they did nothing to stop the new lenders and their new tactics that left so many families without a home.”^29^ She continues, “predatory practices were allowed to continue until the disaster had engulfed white communities, too—and only then, far too late, was it recognized as an emergency.”^29^ If racism had not blinded policymakers and smothered their compassion, people of all races could have been saved from economic harm.

The economists Anne Case and Angus Deaton provide us with another example in their book *Deaths of Despair and the Future of Capitalism*. “Deaths of despair” refers to the rising death rates for the white working class. Case and Deaton argue that this crisis is rooted in the weakened labor market for individuals without college degrees. They also argue that signs of this problem first emerged in black communities 30 years earlier. They state,

deaths of despair among whites would not have happened, or would not have been so severe, without the destruction of the white working class, which, in turn, would not have happened without the failure of the health care system and other problems of the capitalism we have today—*particularly the upward redistribution through manipulation of markets* [emphasis added].^35^

They add,

African Americans have not escaped the crisis but rather experienced their own version first, thirty years earlier…. African Americans, long the least-favored group, were the first to suffer, but less educated whites were next in line.^35^

Again, if racism had not prevented policymakers from addressing labor market problems when it affected black communities 30 years earlier, the white working class would have been spared devastation. As long as we allow racial fictions to lead us to ignore the fact that we, regardless of race, live in the same nation and rely on the same economy, this pattern of preventable economic disasters that hurt all will continue.

A growing number of economic analyses show that an American society committed to equity would be a more prosperous society for all. For example, the economist Lisa Cook's research shows that “riots, lynchings and Jim Crow laws” between 1870 and 1940 cost the nation a number of African American patents for inventions and innovations equivalent to that produced by a medium-sized European country.^36^ Although we are beyond Jim Crow, there is still not equal opportunity to invent and innovate. Looking at America today, the economist Raj Chetty and his colleagues find that “becoming an inventor relies upon two things in America: excelling in math and science *and* having a rich family.”^37^ Children who excel in math and science from low-income or racial minority families are unlikely to become inventors, they add.^37^ Today, class inequality, which is intertwined with racial inequality, stifles U.S. economic innovation and growth by denying opportunity to those who are not rich. The entire country is worse off due to these “lost Einsteins.”^38^

The management consulting firm McKinsey & Company estimates that closing the black–white wealth gap could grow the U.S. economy by $1 to $1.5 trillion by 2028.^39^ Other similar analyses produce similar very large dollar figures.^40,41^ If we are committed to a more equitable America, these additional dollars would benefit Americans of all races. Racism is a powerful force, but we will all gain if we are able to defeat it.
